# Supplementary material for: Occupational Risk for Post-Traumatic Stress Disorder and Trauma-Related Depression: A Systematic Review with Meta-Analysis
Source: Int J Environ Res Public Health. 2020 Dec 14;17(24):9369. doi: 10.3390/ijerph17249369 (PMC7765156; doi:10.3390/ijerph17249369)
Supplement: Supplementary file 1 [file ijerph-17-09369-s001.pdf]

Supplement

Figure S1 *Search string.*
1. In: Pubmed

((((((occupational diseases [MH] OR occupational exposure [MH] OR occupational exposure* [TW] OR "occupational health" OR "occupational medicine" OR work-related OR working environment [TW] OR at work [TW] OR work environment [TW] OR occupations [MH] OR work [MH] OR workplace* [TW] OR workload OR occupation* OR worke* OR work place* [TW] OR work site* [TW] OR job* [TW] OR occupational groups [MH] OR employment OR worksite* OR industry) OR (disaster OR war OR military OR deployment))

AND ((PTSD OR PTSS OR posttraumatic OR post-traumatic OR "stress disorder")))) OR

((((occupational diseases [MH] OR occupational exposure [MH] OR occupational medicine [MH] OR occupational risk [TW] OR occupational hazard [TW] OR (industry [MeSH Terms] mortality [SH]) OR occupational group* [TW] OR work-related OR occupational air pollutants [MH] OR working environment [TW]) OR (disaster OR war OR military OR deployment)))

AND ((depress* OR (affective disorder*)))) AND

((cohort* OR follow-up OR longitudinal OR case-control OR case-referent OR cross-sectional OR "prevalence study" OR population-based OR interview-based OR "registry study"))))

2. In: PILOTS https://www.ptsd.va.gov/professional/pilots-database/

(cohort* OR follow-up OR longitudinal OR case-control OR case-referent OR cross-sectional OR "prevalence study" OR population-based OR interview-based OR "registry study")

AND

((PTSD OR PTSS OR posttraumatic OR post-traumatic OR "stress disorder") OR (depress* OR (affective disorder*)))

Table S1. *Inclusion and exclusion criteria.*

Category	Inclusion criteria	Exclusion criteria
**Population**	General population: employed, both sexes	Children students, unemployed, samples of populations with psychological disorders (i.e. "employed men with mental disorders"), animals
**Exposure**	<u>1. Occupational groups (exposure group 1):</u> Train drivers, soldiers, firefighters, police, rescue workers, emergency doctors, journalists or photographers in war locations, prison personnel, workers in psychiatry<br><br><u>2. Occupational trauma (exposure group 2):</u> Trauma, violence, attack, sexual harassment at the workplace, war deployment	- Neither exposure group 1 or 2<br><br>- Studies with different exposure assessments for diseased and non-diseased participants
**Outcome**	Post-traumatic stress disorder (PTSD), depressive disorder, other affective disorders	- Other mental disorders (i.e. anxiety disorders, adaptive disorders, burn-out)<br><br>- Studies with different outcome assessments for exposed and unexposed participants
**Design**	Cohort studies, case-control studies, cross-sectional studies<br><br>Response ≥ 10%	Qualitative studies, case reports, ecological studies, before-and-after studies, experiments, commentaries, letters, editorials, congress abstracts or posters<br><br>Response <10%, No information on response
**Comparison**	For Exposure Group 1 (Occupational groups):<br><br>a) general employed population<br><br>b) occupational groups in which an average risk can be assumed<br><br>For Exposure Group 2 (Occupational trauma):<br><br>Groups not exposed to occupational trauma (including occupational groups with no occupational trauma, such as soldiers without war deployment)	For Exposure Group 1:<br><br>Studies without comparison groups or or which used comparison groups also exposed to occupational trauma<br><br>For Exposure Group 2:<br><br>- missing comparison group<br><br>- occupational groups with a probable exposure to exposure 1
**Language**	All languages, as long as the abstract was written in German or English	Studies with no English or German abstracts
**Date of Publication**	Since 1994 with the introduction of DSM IV	Before 1994

Table S2 Reasons for exclusion of studies.

Study authors		Study year	Reasons for exclusion: Design_D - Comparison_C-Exposure_E- Outcome_O- Population_P- (see Table S1 in article for details regarding inclusion/exclusion criteria)				
			D	C	E	O	P
S1.	Adams et al.	2016		1			
S2.	Al-Turkait et al.	2008		1			
S3.	Alvarez et al.	2005		1			
S4.	Backholm et al.	2012	1				
S5.	Baggaley et al.	1999		1			
S6.	Ben-Ezra et al.	2013	1				
S7.	Ben-Ezra et al.	2014	1				
S8.	Berninger et al.	2010		1			
S9.	Black et al.	2004		1			
S10.	Blanchard et al.	1996		1			
S11.	Boscarino et al.	1995		1			
S12.	Bramsen et al.	2001		1			
S13.	Chapman et al.	2014	1				
S14.	Cherry et al.	2006		1			
S15.	Clohessy et Ehlers	1999		1			
S16.	da Silva et al.	2016		1			
S17.	Del Ben et al.	2006	1				
S18.	Donoho et al.	2017		1			
S19.	Fullerton et al.	2004		1			
S20.	Gong et al.	2014			1		
S21.	Gonzalez-Ordi et al.	2004	1				
S22.	Goodwin et al.	2015		1			
S23.	Gould et al.	2015		1			
S24.	Gross et al.	2006		1			
S25.	Harvey et al.	2012		1			
S26.	Haslam et al.	2003	1				
S27.	Heymann et al.	2007		1			
S28.	Hotopf et al.	2003		1			
S29.	Ikin et al.	2016		1			
S30.	Iversen et al.	2009	1				
S31.	Jacobson et al.	2012		1			
S32.	Jahnke et al.	2016	1				
S33.	Jenkins et al.	2017	1				

S34.	Johnsen et al.	1997		1			
S35.	Kang et al.	2003		1			
S36.	Kangas et al.	2005			1		
S37.	Kawano et al.	2008	1				
S38.	Kearns et al.	2016		1			
S39.	Kelsall et al.	2014			1		
S40.	Kelsall et al.	2004		1			
S41.	Khashaba et al.	2014	1				
S42.	Koenen et al.	2002		1			
S43.	Koenen et al.	2017			1		
S44.	Koenen et al.	2003		1			
S45.	Lamberg et al.	2003	1				
S46.	Lane et al.	2012		1			
S47.	Larson et al.	2008		1			
S48.	Lehavot et al.	2012		1			
S49.	Lehavot et al.	2018a	1				
S50.	Leovat et al.	2013		1			
S51.	Li et al.	2011		1			
S52.	Li et al.	2006		1			
S53.	Limosin et al.	2006	1				
S54.	Liu et al.	2013		1			
S55.	Lu et al.	2014		1			
S56.	Luce Firth-Cozens et al.	2002		1			
S57.	MacGregor et al.	2015		1			
S58.	Maguen et al.	2009			1		
S59.	Magruder et al.	2015		1			
S60.	Marsh et al.	2009	1				
S61.	McKenzie et al.	2004		1			
S62.	McMahon et al.	2001				1	
S63.	Milligan-Saville et al.	2018	1				
S64.	Mion et al.	2013				1	
S65.	Morales et al.	2012		1			
S66.	Morren et al.	2005		1			
S67.	Munson et al.	2000	1				
S68.	Murdoch et al.	2006		1			
S69.	Murdoch et al.	2007	1				
S70.	Nelson et al.	2011		1			
S71.	Nguyen et al.	2013				1	
S72.	Niedhammer et al.	2015			1		
S73.	Niedhammer et al.	2016			1		
S74.	Osório et al.	2017		1			
S75.	O'Toole et al.	2009		1			
S76.	O'Toole et al.	1998		1			

S77.	O'Toole et al.	1999		1			
S78.	Park et al.	2016			1		
S79.	Polusny et al.	2011	1				
S80.	Rodríguez-Rey et al.	2018			1		
S81.	Rona et al.	2007		1			
S82.	Roy-Byrne et al.	2004		1			
S83.	Schwartz et al.	1997		1			
S84.	Scott et al.	2014		1			
S85.	Seelig et al.	2012		1			
S86.	Shi et al.	2017	1				
S87.	Simmons et al.	2004		1			
S88.	Slottje et al.	2008		1			
S89.	Smith et al.	2009		1			
S90.	Solomon et al.	1996	1				
S91.	Stein et al.	2005		1			
S92.	Street et al.	2008			1		
S93.	Stretch et al.	1996		1			
S94.	Taal et al.	2014		1			
S95.	Theorell et al.	1994		1			
S96.	Toomey et al.	2009			1		
S97.	Unwin et al.	1999		1			
S98.	Vaingankar et al.	2015			1		
S99.	Vanderploeg et al.	2012		1			
S100.	Vasterling et al.	2010		1			
S101.	Vedantham et al.	2001				1	
S102.	Whyman et al.	2011			1		
S103.	Wieclaw et al.	2005			1		
S104.	Wieclaw et al.	2006			1		
S105.	Witteveen et al.	2007	1				
S106.	Wolfe et al.	1999		1			
S107.	Yip et al.	2016		1			
S108.	Zerach et al.	2015	1				
S109.	Zhen et al.	2012	1				
S110.	Ziobrowski et al.	2017			1		

Table S3 *Characteristics of included studies.*

No.	First sauthor, publication year	Study region	Study design	Population			Exposure,<br>duration of employment,<br>job duties	Outcome measurement, Institution, Finance
				**Sample population**	**No. of exposed/ No. of cases<br>No. of unexposed / No. of controls<br>(Response rate, age)**	**Time of recruitment**		
1	*Ben Ezra et al. 2011*	*Israel*	*Cross sectional study*	*Random samples<br>Two separated groups for study 1 and 2 physicians and nurses*	*No. of exposed:*<br>*67, Study 1 // 57, Study 2 (physicians and nurses)*<br>*No. of unexposed:*<br>*74, Study 1 // 50, Study 2 (physicians and nurses)*<br>*Response rate:*<br>*85% exp. 90% unexp., Study 1*<br>*82% exp. 87% unexp., Study 2*<br>*Age (mean ± SD):*<br>*Study 1*<br>*40.67 (± 10.11) years, exp.*<br>*38.46 (± 9.45) years, unexp.*<br>*Study 2*<br>*exp. 40.65 (± 10.63) years,*<br>*unexp. 37.30 (± 9.07) years*<br>*Gender:*<br>*Study 1*<br>*50% female 50% male, exp.*<br>*51% female 49% male, unexp.*<br>*Study 1*<br>*46% female 54% male, exp,*<br>*40% female 60% male, unexp.*	*January 2009 study 1 (during Gaza war)*<br>*July 2009 study 2 (six month after Gaza war)*	*Exposure:*<br>*exposed to war-related stress Treatment of war victims (soldiers and civilians)*<br>*Duration of employment:*<br>*Study 1: During war*<br>*Study 2: Six months after war*<br>*Job duties:*<br>*working in hospital*	*PTSD:*<br>*Impact of Event Scale-revisited (IES-R),22 items, three subscales (intrusion, avoidance, hyperarousal), Likert scale 0-4 (range 0-88), referring to the past 7 days*<br>*Depression:*<br>*Centre for Epidemiologic Studies Depression Scale (CES-D), 20 items, four subscales (negative affect, positive affect, somatic symptoms, interpersonal problems), Likert scale 0-3, (range 0-60), referring to the last 7 days,*<br><br>*Blinded application of questionnaire*<br>*☒no, interview*<br>*☐not reported*<br>*☐yes:*<br>*Institution: School of Social Work, Ariel University center of Samaria, Ariel*<br>*Finance: n.I.*<br>*Institution of author/coauthor: Department of Gerontology, University of Hafia, Department of Psychology, Trinity College Dublin, Department of Psychology, Tel Aviv University*
2	Berg et al. 2006	Nor-way	Cross sectional study	Police	No. of exposed:<br>3,272 members of the Norwegian police union<br>No. of unexposed:<br>61,216 persons in the general population in Nord-Trøndelag<br>Response rate:<br>51.0 % members of the Norwegian police union<br>71.3 % general population<br>Age (mean)[1]:	12/2000 police<br>n.I. in the control group	Exposure:<br>N.I.<br>Duration of employment:<br>N.I.<br>Job duties:<br>Police: investigation uniformed policing, and administration	PTSD:<br>Not asked<br>Depression:<br>Hospital Anxiety and Depression Scale (HADS)<br>Blinded application of questionnaire<br>☐no, interview<br>☐not reported

					38.92 years members of the Norwegian police union, SD n.I.<br>N.I. to age of the control group<br>Gender:<br>15.7 % female 84.3 % male members of police<br>n.I. to gender of the control group			☒yes: police, n.I. according to the control group<br>Institution: University of Oslo<br>Finance: Norwegian Department of Justice, Norwegian Foundation for Health and Rehabilitation, the Norwegian Institute of Public Health
3	Cothereau et al.<br>2004	France	Cross sectional study with re-examina-tion of the exposed subgroup immediately after the accident and 3 months, 1, 2 and 3 years later	Train drivers	No. of exposed:<br>202 train drivers exposed „person under train“,<br>No. of unexposed:<br>186 train drivers non-exposed<br>Response rate:<br>93% exp. and unexp.<br>Age (mean ± SD):<br>39 (± 6.5) years, exp.<br>39.1 (± 6.3) years, unexp.<br>Gender:<br>Only male drivers	30 May 1996-30 September 2000<br>Evaluated immediately after accident, again three months, and one, two and three years after accident	Exposure:<br>person under train accident<br>Duration of employment:<br>N.I.<br>Job duties:<br>Driving train	PTSD:<br>MINI international psychiatric interview<br>Depression:<br>MINI international psychiatric interview (MINI, DSM IV), General Health Questionnaire (GHQ-28), 28 item, threshold GHQ score of 5 (=indicator of psychiatric disorder)<br><br>Blinded application of questionnaire<br>☐yes<br>☒no, self-questionnaire (GHQ) given by the occupational physicians involved in the study, Interview (MINI) by the occupational physicians involved in the study<br>☐not reported<br>Institution: Société Nationale des Chemins de Fer Français (SNCF), Direction des Ressources Humaines, Département des Services Médicaux, Paris<br>Finance:<br>Institution of author/coauthor: Hospital La Pitié-Salpètrière, Paris, Hopital Chenevier, Créteil
4	Darves-Bornoz et al.<br>2008	Belgium, France,	Cross sectional study	The European study of the Epidemiolog	No. of exposed:<br>214 persons with combat experience<br>No. of unexposed:<br>8,582 persons without combat experience	January 2001-August 2003	Exposure:<br>combat experience<br>Duration of employment:<br>n.I.	PTSD:<br>WHO composite international diagnostic interview (CIDI 2000, DSM-IV), 28 types of

		Netherlands, Germany, Italy, Spain,		y of Mental Disorder Survey (ESEMeD)<br>A personal household survey, population based study	<u>Response rate:</u><br>61.2%<br>N.I. according to response rate in persons exposed and unexposed to combat experience<br><u>Age (mean):</u><br>47 years (CI 46.8-47.4)<br>N.I. according to age in persons exposed and unexposed to combat experience<br><u>Gender:</u><br>Not available		<u>Job duties:</u><br>n.I.	PTEs, referring in the year preceding the interview<br><u>Depression:</u><br>Not asked<br><br>Blinded application of questionnaire<br>☐yes<br>☒no trained interviewer without clinical experience<br>☐not reported<br>Institution: Hospital Henri-Ey, Paris<br>Finance: funded by the European Commission, local agencies and GlaxoSmithKline<br>Institution of author/co-author: WHO World Mental Health Survey Initiative
5	Feinstein et al. 2002	All over the world	Cross sectional study	War journalists	<u>No. of exposed:</u><br>140 war journalists<br><u>No. of unexposed:</u><br>107 journalists without war experience<br><u>Response rate:</u><br>82.8% exp.<br>79.9% unexp.<br><u>Age (mean ± SD):</u><br>39.2 ± 6.3 years, exp.<br>39.0 ± 8.2 years, unexp.<br><u>Gender:</u><br>21.4% female 78.6 % male, exp.<br>29.0% female 71.0 % male, unexp.	N.I.	<u>Exposure:</u><br>war experience<br><u>Duration of employment:</u><br>war journalists (exp.) 15.6 ± 6.8 years<br>controls (unexp.) 15.5 ± 8.5 years<br><u>Job duties:</u><br>journalism	<u>PTSD:</u><br>impact of event scale-revised (IES-R), DSM-IV (intrusive, avoidance, hyperarousal)<br><u>Depression:</u><br>Beck depression inventory II (BDI II) and depression subscale of General health questionnaire (GHQ 28), Clinical interview for axis I DSM IV disorders (SCID) in 28 war journalists and 19 controls<br>Blinded application of questionnaire Self-report questionnaire<br>☒yes all participants: confidential identifications number to enter a web side<br>☒no 20% sample: direct interview<br>☐not reported<br>Institution: Department of Psychiatry, University of Toronto and Sunnybrook and Women`s College Health Sciences Centre,

								Finance: Freedom Forum and Guggenheim Foundation<br>Institution of author/co-author: Freedom Forum and Guggenheim Foundation
6	Feinstein 2012	Mexic o	Cross sectional study	Journalists	No. of exposed:<br>26 journalists threatened by drug cartels and stopped reporting on drug-related issues, exp. I<br>61 journalists threatened by drug cartels and continued reporting on drug-related issues,exp.II<br>No. of unexposed:<br>17 journalists not threatened by drug cartels<br>Response rate:<br>80.6 %<br>N.I. according to response rate in the aforementioned subgroups<br>Age (mean ± SD):<br>42.5 ± 10.2 years<br>N.I. according to age in the aforementioned subgroups<br>Gender:<br>32.7% female, 67.3 % male<br>N.I. according to gender in the aforementioned subgroups	N.I.	Exposure:<br>threatened by drug cartels and stopped reporting on drug-related issues<br>Duration of employment:<br>n.I.<br>Job duties:<br>journalism	PTSD:<br>impact of event scale-revised (IES-R), 22 item, three subscales (Intrusion, Avoidance, Hyperarousal)<br>Depression:<br>Beck depression inventory II (BDI II) and depression subscale of General health questionnaire (GHQ 28)<br><br>Blinded application of questionnaire'<br>☐yes<br>☐no<br>☒not reported<br>Institution: Department of Psychiatry, University of Toronto and Sunnybrook and Women`s College Health Sciences Centre<br>Finance: not reported<br>Institution of author/co-author:, no co-author
7	Hotopf et al.<br>2006	United Kingd om (UK)	Cross sectional, author means cohort, but no incident data	UK armed forces	No. of exposed:<br>1,290 regulars and reservists deployed to Iraq war<br>No. of unexposed:<br>1,051 regulars and reservists not deployed to any war<br>Response rate:<br>62.3 % exp.<br>56.3 % unexp.<br>Age:<br>>25-<50 years, SD n.I.<br>N.I. according to age (mean) in persons exposed and unexposed to combat experience<br>Gender:<br>Only male soldiers	1-4/2003 (deployed on Operation TELIC1)<br>3/2003 (ERAQ comparison population)<br><br>Questionnaire 6/2004-3/2006	Exposure:<br>Iraq war combat experience<br>Duration of employment:<br>n.I.<br>Job duties:<br>n.I.	PTSD:<br>Checklist (PCL-C)<br>Depression:<br>Not asked<br><br>Blinded application of questionnaire'<br>☐yes<br>☐no<br>☒not reported<br>Institution: King´s Centre of military< Health Research<br>Finance: UK Ministry of Defence<br>Institution of author/co-author: Academy Centre for Defence Mental Health, Department of Biostatistics.

								Institute of Psychiatry, School of Social Science and Public Policy and Division of Asthma, Allergy and Lung Biology, Kings College London, Royal Hospital Haslar, Gosport, UK Ministry of Defence
8	Huizink et al. 2006	Ne-ther-land	Cross sectional study	Firefighters and policemen involved in an aircraft crash October 4th 1992	<u>No. of exposed:</u><br>334 firefighters, 834 police officers exposed to the aircraft crash<br><u>No. of unexposed:</u><br>194 firefighters, 634 police officers not exposed to the aircraft crash<br><u>Response rate:</u><br>70 % n.I. according to response rate in the aforementioned subgroups<br><u>Age (mean ± SD):</u><br>51.4 ± 5.9 years exposed firefighter<br>44.0 ± 6.2 years exposed police officers<br>38.8 ± 9.1 years unexposed firefighters<br>44.8 ± 7.0 years unexposed police officers<br><u>Gender:</u><br>100 % male firefighters,<br>11.5 % female, 88.5 % male exposed police officers<br>15.1 % female, 84.9 % male unexposed police officers	8.5 years (on average) post disaster	<u>Exposure:</u><br>Handling of an aircraft crash<br><u>Duration of employment:</u><br>n.I.<br><u>Job duties:</u><br>n.I.	<u>PTSD:</u><br>Self-rating inventory for posttraumatic stress disorder<br><br><u>Depression:</u><br>Not asked<br><br>Blinded application of questionnaire'<br>☐yes<br>☒no<br>☐not reported<br>Institution: Erasmus Medical Center of Child and Adolescent Psychiatry<br>Finance/Funding: Ministry of Health, Welfare and Sports, City of Amsterdam, regional police force Amsterdam-Amstelland, KLM Royal Dutch Airline<br>Institution of author/co-author:
9	Kim et al. 2013	Repub lic of Korea n	Cross sectional study	Subway drivers	<u>No. of exposed:</u><br>266 underground drivers with person under train accident<br><u>No. of unexposed:</u><br>560 underground drivers without person under train accident<br><u>Response rate:</u><br>86.7%<br>N.I. according to response rate in the aforementioned subgroups	3-8/2007	<u>Exposure:</u><br>Persons-under-train (PUT) experience<br>Whether victims died, how many PUTs the drivers experienced<br><u>Duration of employment:</u><br>Table 1 Years<br><u>Job duties:</u><br>Subway drivers	<u>PTSD:</u><br>Korean version of the Composite International Diagnostic Interview (K-CIDI 2.1), DSM IV<br><u>Depression:</u><br>See above<br><br>Blinded application of questionnaire'<br>☐yes

					Age (mean):<br>37.9 years, SD n.I.<br>N.I. according to age in the aforementioned subgroups<br>Gender:<br>Only male persons			☒no, interviewer<br>☐not reported<br>Institution: Department of Preventive Medicine, Catholic Industrial Medical Centre, College of Medicine<br>Finance: Korea Healthcare Technology R&D Project, Ministry for Health and Welfare, Republic of Korea, Seoul Metropolitan Rapid Transit Corporation<br>Institution of author/co-author: Division of Occupational and Environmental Health, Centre of occupational und Environmental Health, Clinical Research Coordinating, Department of Preventive Medicine, Department of Psychiatry, Scholl of Medicine, Kangwon National University
10	Kim et al. 2014	Republic of Korean	Cross sectional study	Subway drivers	No. of exposed:<br>N.I.<br>No. of unexposed:<br>N.I.<br>Response rate:<br>99.7 %<br>N.I. according to response rate in the aforementioned subgroups<br>Age (mean):<br>43.7 years, SD n.I.<br>N.I. according to age in the aforementioned subgroups<br>Gender:<br>Only male persons		Exposure:<br>Questionnaire<br>Severty of victims<br>Duration of employment:<br>Number and time of Persons-under-train (PUT) experience<br>Job duties:<br>n.I.	PTSD:<br>Korean version of the Composite International Diagnostic Interview (K-CIDI 2.1), DSM IV<br>Depression:<br>Composite International Diagnostic Interview 2.1<br>Blinded application of questionnaire’<br>☐yes<br>☒no, trained interviewers<br>☐not reported<br>Institution: Department of Preventive Medicine, Catholic Industrial Medical Centre, College of Medicine<br>Finance: n.I.<br>Institution of author/co-author: Division of Occupational and Environmental Health, Centre of occupational and Environmental Health, Clinical Research Coordinating, Department of

								Preventive Medicine, Department of Psychiatry, Scholl of Medicine, Kangwon National University
11	Kline et al. 2010	USA	Cross sectional study	National Guard Troops	No. of exposed:<br>625 National Guard Troops with one or more previous deployments to the war in Afghanistan or Iraq<br>No. of unexposed:<br>1,910 National Guard Troops without previous deployments to war in Afghanistan or Iraq<br>Response rate:<br>88.9 %<br>N.I. according to response rate in persons exposed and unexposed to combat experience<br>Age (mean ± SD):<br>33.2 ± 8.9 years, exp.<br>29.0 ± 9.3 years, unexp.<br>Gender:<br>14.5% female, 85.5 % male, exp.<br>11% female, 89% male, unexp.	November 2007 – May 2008	Exposure:<br>Deployment to the war in Afghanistan or Iraq<br>Duration of employment:<br>n.I.<br>Job duties:<br>n.I.	PTSD:<br>Checklist (PCL)<br>Depression:<br>depression scale of the Patient Health Questionnaire., DSM IV<br><br>Blinded application of questionnaire'<br>☒yes, anonymous, self-administered surveys, participation was not mandatory, no monetary incentives<br>☐no<br>☐not reported<br>Institution: Department of Veterans Affairs, New Jersey Health Care System<br>Finance: New Jersey Department of Military and Veterans Affairs<br>Institution of author/coauthor: University of Medicine and Dentistry of New Jersey
12	Lam et al. 1999	Aus-tralia	Cross sectional study	Nurses of four major hospitals	No. of exposed:<br>257 nurses exposed to traumatic situations.<br>No. of unexposed:<br>57 nurses unexposed to traumatic situations (in analysis not included).<br>Response rate:<br>86.7%<br>N.I. according to response rate in the aforementioned subgroups<br>Age (mean ± SD):<br>37.9 ± 9.9 years<br>N.I. according to age in the aforementioned subgroups<br>Gender:<br>93% female, 7% male<br>N.I. according to gender and response rate in the aforementioned subgroups	N.I.	Exposure:<br>Hospital Trauma exposure Checklist (HTEC)<br>High exposure was defined as above the medium score and low exposure as below the medium score. The analysis compared only high and low exposed nurses. The exact number of high and low exposed nurses is not described.<br>Duration of employment:<br>Mean duration of employment 5.1 (± 3.8) years<br>Job duties:<br>working in hospital	PTSD:<br>not asked<br><br>Depression:<br>Beck´s Depression Inventory-Revised (BDI-R)<br><br>Blinded application of questionnaire'<br>☐yes<br>☐no<br>☒not reported<br>Institution: Trauma research unit, New Children's Hospital, Westhead, Australia<br>Finance: Health and Family Services Research and Development Grant, Commonwealth Department of Health and Family Services, Australia

13	Lehavot et al. 2018	USA	Cross sectional study	Representative sample of US residents ≥ 18 years: veterans and civilians (NESARC-III)	No. of exposed:<br>59 female and 669 male veterans exposed to combat or war deployment.<br>No. of unexposed:<br>320 female and 2,071 male veterans not exposed to combat or war deployment.<br>Response rate:<br>60.1%<br>N.I. according to response rate in persons exposed and unexposed to combat experience<br>Age mean (± SD):<br>51.94[1] years (female veterans),<br>61.73[1] years (male veterans),<br>47.36[1] years (civilians)<br>SD n.I., n.I. according to age in persons exposed and unexposed to combat experience<br>Gender:<br>12.1% female, 87.9% male, veterans:<br>60.7% female, 39.3% male, civilians:<br>N.I. according to gender in persons exposed and unexposed to combat experience	2012-2013	Exposure:<br>exposure to combat or war zone<br>Duration of employment:<br>n.I.<br>Job duties:<br>n.I.	PTSD:<br>Alcohol Use Disorder and Associated Disabilities Interview Schedule-DSM-5 Version (AUDADIS-5)<br>(DSM V)<br>Depression:<br>Not asked<br>Blinded application of questionnaires'<br>☐yes<br>☐no<br>☒not reported<br>Institution: Veterans Administration, Seattle, USA<br>Finance: Veterans Administration Career Development Awards
14	Levin-Rector et al. 2018	USA	Cohort study	All service members who accessed into the US Army or US Marine Corps	773,359 army soldiers<br>332,093 marines<br>No. of exposed:<br>N.I. according to the number of persons exposed to combat experience<br>No. of unexposed:<br>N.I. according to the number of persons unexposed to combat experience<br>Response rate:<br>95.2 % army soldiers<br>91.8 % marines<br>N.I. according to response rate in persons exposed and unexposed to combat experience<br>Age (mean ± SD):<br>21.7 ± 3.9 years, army soldiers<br>20.0 ± 2.1 years, marines<br>N.I. according to age in persons exposed and unexposed to combat experience<br>Gender:<br>17.4% female, 82.6 % male, army soldiers<br>7.4% female, 92.6 % male, marines	January 2001 – December 2011	Exposure:<br>Combat experience<br>Duration of employment:<br>N.I.<br>Job duties:<br>Categorized into six occupational groups	PTSD:<br>Inpatient or outpatient ICD-9-diagnosis of PTSD according the archival medical personnel system (CHAMPS)<br>Depression:<br>Inpatient or outpatient ICD-9-diagnosis of depression according the archival medical personnel system (CHAMPS)<br><br>Blinded application of questionnaire'<br>☐yes<br>☒no<br>☐not reported<br>Institution: RTI International, Research Triangle Park, NC, USA<br>Finance: US Army Medical Research and Materiel Command

					N.I. according to gender in persons exposed and unexposed to combat experience			
15	Luce et al. 2002	Nor-thern Ireland	Cross sectional study	Health service staff working closest to Omagh	<u>No. of exposed:</u><br>536 (all together) health service staff members reported being exposed:<br>309 exposed purely professionally<br>130 exposed as a civilian<br>97 exposed both professional and civilian<br><u>No. of unexposed:</u><br>528 health service staff members reported being unexposed<br><u>Response rate:</u><br>35% (all together)<br>30% target population<br>18% domestic/manual worker<br>50% PAMs<br>48% managers<br>N.I. according to response rate in persons exposed and unexposed<br><u>Age (mean ± SD):</u><br>N.I.<br><u>Gender:</u><br>N.I.	Four months after Omagh bombing with a reminder six months after the bombing August 15th , 1998	<u>Exposure:</u><br>Omagh Bomb<br><u>Duration of employment:</u><br>N.I.<br><u>Job duties:</u><br>Seven scores of experiences in exposure	<u>PTSD:</u><br>Self-report Posttraumatic Stress Disorder Symptom Scale (PSS-SR), DSM-III_R<br><u>Depression:</u><br>Not asked<br><br>Blinded application of questionnaire'<br>☐yes<br>☒no<br>☐not reported<br>Institution: University Newcastle upon Tyne, United Kingdom<br>Finance: n.I.<br>Institution of author/co-author:
16	Madsen et al. 2010	Den-mark	Cohort study	Merged data: Danish work environment cohort study (DWECS) and the register of medicinal product statistics	<u>No. of exposed:</u><br>312 exposed to threats, 115 exposed to violence<br><u>No. of unexposed:</u><br>4,646 unexposed to threats, 4,843 unexposed to violence<br><u>Response rate:</u><br>75%<br>N.I. according to response rate in the aforementioned subgroups<br><u>Age (mean):</u><br>40.3 years, SD n.I.<br>N.I. according to response rate in the aforementioned subgroups<br><u>Gender:</u><br>48.5% female, 51.5% male<br>N.I. according to response rate in the aforementioned subgroups	2000	<u>Exposure:</u><br>threats of violence or physical violence in workplace during the past 12 months.<br><u>Duration of employment:</u><br>n.I.<br><u>Job duties:</u><br>Classification of occupation was based on a modified version of the International Standard of Classification of Occupations (ISCO-68)	<u>PTSD:</u><br>not asked<br><u>Depression:</u><br>At baseline 5-item mental health inventory of the 36-item short-form (SF-36) was used, persons scoring ≤ 52 points (severe depressive symptoms) were excluded, purchase of antidepressants was registered with the register of medicinal product statistics during the 5-year follow-up.<br><br>Blinded application of questionnaire'<br>☐yes<br>☐no<br>☒not reported

								Institution: National Research Centre for the Working Environment, Copenhagen, Denmark<br>Finance: Danish Working Environment Authority
17	Magruder et al. 2005	USA	Cross sectional study	Veterans attending four primary care clinics	No. of exposed:<br>420 veterans, served in war zone<br>No. of unexposed:<br>465 veterans, did not serve in a war zone<br>Response rate:<br>74.1%<br>N.I. according to response rate in persons exposed and unexposed to combat experience<br>Age (mean ± SD):<br>60.9 ±12.1 years<br>N.I. according to age in persons exposed and unexposed to combat experience<br>Gender:<br>7.1% female, 92.1% male<br>N.I. according to gender in persons exposed and unexposed to combat experience	1999	Exposure:<br>War deployment according Trauma Assessment for Adults Questionnaire<br>Duration of employment:<br>n.I.<br>Job duties:<br>n.I.	PTSD:<br>DSM-IV, Trauma Assessment for Adults Questionnaire (Clinician Administered PTSD Scale- CAPS).<br>Mini International Neuropsychiatric Interview (MINI)<br>Depression:<br>Not asked<br><br>Blinded application of questionnaire'<br>☐yes<br>☒no<br>☐not reported<br>Institution: Ralph H. Johnson Veterans Affairs Medical Centre Charleston, SC, USA<br>Finance: Veterans Affairs Health Services Research and Development Program
18	Niedham-mer et al. 2015	France	Cross sectional study	Population based study (SUMER)	No. of exposed:<br>375 women exposed to physical violence or sexual assault<br>402 men exposed to physical violence or sexual assault<br>No. of unexposed:<br>19,112 women unexposed to physical violence or sexual assault<br>25,813 men unexposed to physical violence or sexual assault<br>Response rate:<br>87%<br>N.I. according to response rate in the aforementioned subgroups<br>Age (mean[1] ± SD):<br>40.46 years[1] female, 40.06 years[1] male	2010	Exposure:<br>physical violence or sexual assault assessed by questionnaire<br>Duration of employment:<br>n.I.<br>Job duties:<br>n.I.	PTSD:<br>Not asked<br>Depression:<br>Hospital Anxiety and Depression (HAD) scale<br>Blinded application of questionnaire'<br>☐yes<br>☐no<br>☒not reported<br>Institution: Institute of Epidemiology and Public Health INSERM, Paris<br>Finance: French ministry of labour

					SD n.I.<br>N.I. according to age in the aforementioned subgroups<br><u>Gender:</u><br>42.76% female, 54.24% male<br>N.I. according to gender in the aforementioned subgroups			
19	Oenning et al. 2018	Brazil	Cross sectional study	Brazilian National Health Survey	<u>No. of exposed:</u><br>231 women exposed to workplace violence<br>250 men exposed to workplace violence<br><u>No. of unexposed:</u><br>16,771 women not exposed to workplace violence<br>19,200 men not exposed to workplace violence<br><u>Response rate:</u><br>91.9%<br>N.I. according to response rate in the aforementioned subgroups<br><u>Age (mean[1] ± SD):</u><br>38.95 years[1] female, 39.68 years[1] male,<br>SD n.I.<br><u>Gender:</u><br>48.02% female, 51.98% male	August 2013-February 2014	<u>Exposure:</u><br>Workplace violence according to questionnaire<br><u>Duration of employment:</u><br>N.I.<br><u>Job duties:</u><br>N.I.	<u>PTSD:</u><br>Not asked<br><u>Depression:</u><br>Major depressive disorder (MDD),Diagnostic and Statistical Manual of Mental Disorders, 4th Edition (DSM-IV), PHQ-9<br>Blinded application of questionnaire'<br>☐yes<br>☒no trained interviewers<br>☐not reported<br>Institution: Epidemiology Program, University Porto Alegre, Brazil<br>Finance: Coordination of Improvement of Higher Level Personnel, Brazil
20	Opie et al. 2010	Austra lia	Cross sectional study	Nurses working in very remote regions across Australia	<u>No. of exposed:</u><br>79 nurses exposed to sexual harassment at work<br>9 nurses exposed to sexual abuse/assault work<br><u>No. of unexposed:</u><br>270 nurses not exposed to sexual harassment at work<br>340 nurses not exposed to sexual abuse/assault at work<br><u>Response rate:</u><br>34,6%, n.I. according to response rate in the aforementioned subgroups<br><u>Age (mean ± SD):</u><br>44 years ± 11years, n.I. according to age in the aforementioned subgroups<br><u>Gender:</u><br>88.5% female, 11.5% male	2008	<u>Exposure:</u> sexual harassment or sexual abuse/assault according to a questionnaire<br><u>Duration of employment:</u><br>n.I.<br><u>Job duties:</u><br>Nurses	<u>PTSD:</u> PTSD checklist (PCL)<br><br><u>Depression:</u><br>Not asked<br>Blinded application of questionnaire'<br>☐yes<br>☐no<br>☒not reported<br>Institution: University of South Australia<br>Finance: n.I.

21	Park 2011	USA	Cross sectional study	Nurses 5 years after hurricane Katrina	<u>No. of exposed:</u><br>76 nurses exposed to hurricane Katrina<br><u>No. of unexposed:</u><br>32 nurses not exposed to hurricane Katrina<br><u>Response rate:</u><br>11.9% n.I. according to response rate in the aforementioned subgroups<br><u>Age (mean ± SD):</u><br>50.0 years ± 11.8 years<br><u>Gender:</u><br>93.5 % female, 6.5 % male	2011	<u>Exposure:</u><br>Involved as nurse in hurricane Katrina, trauma assessed by the trauma exposure severity scale<br><u>Duration of employment:</u><br>n.I.<br><u>Job duties:</u><br>Nurse	<u>PTSD: I</u><br>Impact of event scale (IES)<br><br><u>Depression:</u><br>Not asked<br>Blinded application of questionnaire'<br>☐yes<br>☒no<br>☐not reported<br>Institution: Georgia State University<br>Finance: No finance
22	Proctor et al. 1998	USA	Cross sectional study	Soldiers in the Persian Gulf War (January-April 1991)	<u>No. of exposed:</u><br>206 soldiers exposed to Persian Gulf War<br><u>No. of unexposed:</u><br>48 soldiers deployed in Germany not exposed to war deployment<br><u>Response rate:</u><br>85% (Devens cohort),<br>58% (New Orleans cohort)<br>85% (Germany cohort)<br><u>Age (mean ± SD):</u><br>34.7 years ± 9.3 years (Devens cohort),<br>34.3 years ± 8.8 years (New Orleans cohort), 41.0 years ± 9.0 years (Germany cohort)<br><u>Gender:</u><br>46.2% female, 53.8% male (Devens cohort),<br>48.5% female, 51.5% male (New Orleans cohort),<br>14.6% female, 85.4% male (Germany cohort)	1994-1996	<u>Exposure:</u><br>war related traumata according the expanded combat exposure scale (CES)<br><u>Duration of deployment:</u><br>n.I.<br><u>Job duties:</u><br>n.I.	<u>PTSD:</u><br>Clinician administered scale for PTSD (CAPS) in 75% of subjects and Mississippi PTSD scale in 99% of subjects.<br><br><u>Depression:</u><br>Not asked<br><br><u>Blinded application of questionnaire'</u><br><u>☐yes</u><br><u>☒no</u><br><u>☐not reported</u><br>Institution: Boston University<br>Finance: US-Department of Veterans Affairs
23	Rosellini et al. 2017	USA	Prospective cohort study	All regular female US army soldiers	<u>No. of exposed:</u><br>4,238 female soldiers exposed to sexual assault<br><u>No. of unexposed:</u><br>21,190 matched female soldiers not exposed to sexual assault)<br><u>Response rate:</u><br>100%<br><u>Age</u>:<br>age-matched<br><u>Gender:</u><br>All female	2004-2009	<u>Exposure:</u><br>Sexual assault victimisation according 5 criminal justice data systems<br><u>Duration of employment:</u><br>n.I.<br><u>Job duties:</u><br>n.I.	<u>PTSD: </u>Treatment because of PTSD 12 month subsequent each sexual assault according the historical administrative data system of the US army<br><br><u>Depression:</u><br>Not asked<br><br>Blinded application of questionnaire'<br>☒yes<br>☐no<br>☐not reported

								Institution: Harvard Medical School, Boston, M.A., USA<br>Finance: National Institute of mental Health
24	Sareen et al. 2007	Canadian	Cross-sectional Study	(Canadian forces target) Population-based survey (Canadian Community Health Survey Cycle 1.2 Canadian Forces Supplement, CCHS-CFS)	No. of exposed²:<br>1117 forces exposed to combat<br>921 forces exposed witnessing atrocities or massacres<br>2,598 peacekeeping operations<br>No. of unexposed:<br>7,324 forces not exposed to combat<br>7,526 forces not exposed witnessing atrocities or massacres<br>5,843not exposed to peacekeeping operations<br>Response rate:<br>79,5% regular force member<br>83,5% reserve force member<br>n.I. according to response rate in the aforementioned subgroups<br><br>Age (mean¹ ± SD):<br>33,97 years¹, SD n.I., n.I. according to age in the aforementioned subgroups<br>Gender:<br>14.7% female, 85.3% male<br>n.I. according to gender in the aforementioned subgroups	2002	Exposure<br>to combat, witnessing atrocities/massacres or peacekeeping operations according to a 28-item trauma exposure questionnaire<br><br>Duration of employment:<br>n.I.<br><br>Job duties:<br>soldiers	PTSD:<br>DSM-IV, ICD-10<br>World Mental Health version of the World Health Organisation Composite International Diagnostic Interview (WHO CIDI version 2.1)<br>Depression:<br>World Mental Health version of the World Health Organisation Composite International Diagnostic Interview<br><br>Blinded application of questionnaire'<br>☐yes<br>☒no trained interviewers<br>☐not reported<br>Institution: Department of Psychiatry, Community Health Sciences and Psychology, University of Manitoba, Department od Psychiatry and Family and Preventive Medicine, University of California San Diego, Veterans Affairs San Diego Healthcare System, San Diego, Department of Psychiatry, University of Melbourne, Melbourne, Australia, Anxiety and Illness Behaviours Laboratory and Traumatic Stress Group, University of Regina, Regina, Saskatchewan<br>Finance/Funding: Canadian Institutes of Health Research (CIHR), Manitoba Health Research Council Establishment and CIHR New Investigator grants

25	Sterud et al. 2008	Norway	Cross sectional study	Operational ambulance personnel, population-based survey	<u>No. of exposed:</u><br>1,180 ambulance personnel<br><u>No. of unexposed:</u><br>31,987 working people<br><u>Response rate:</u><br>41% (range 31%-59%) exposed group (ambulance personnel)<br>71% control group<br><u>Age (mean ± SD):</u><br>36.8 ± 9.3 years exp.<br>41.1 ± 9.3 years unexp.<br><u>Gender:</u><br>23.2% female, 76.8% male, exp. (ambulance personnel)<br>53% female, 47% male unexp.	Ambulance workers: 2005<br>Population controls: 1995-1997	<u>Exposure:</u><br>N.I. to specific traumata as ambulance worker<br><u>Duration of employment:</u><br>n.I.<br><u>Job duties:</u><br>Personnel ambulance	<u>PTSD:</u><br>Not asked<br><u>Depression:</u><br>Hospital Anxiety and Depression scale (HADS), depression subscale with seven items<br>Blinded application of questionnaire'<br>☐yes<br>☒no<br>☐not reported<br>Institution: Department of Behaviour Sciences in Medicine, University of Oslo<br>Finance: Declaring of no competing interests, The Eastern Norway Regional Health Authority, The Lardal Foundation for Acute Medicine
26	Thomas et al. 2017	USA	Cross sectional study	Veterans, Nationally representative Survey	<u>No. of exposed:</u><br>564 (38.4%) US veterans, who did ever serve in a combat or war zone<br><u>No. of unexposed:</u><br>916 (61.6%) US veterans, who did never serve in a combat or war zone<br><u>Response rate:</u><br>92.6% n.I. according to response rate in the aforementioned subgroups<br><u>Age (mean ± SD):</u><br>59.0 ± 16.6 years exp.<br>61.3 ± 14.3 years unexp.<br><u>Gender:</u><br>7.0% female 93.0% male exp.<br>12.4% female 87.6% male unexp.	September-October 2013	<u>Exposure:</u><br>Soldiers in combat<br>43.6% served during Vietnam War<br>Median year of last deployment was 1971<br>Median number of deployment was 1<br><u>Duration of employment:</u><br>N.I.<br><u>Job duties:</u><br>Soldiers	<u>PTSD:</u><br>Posttraumatic Stress Disorder Checklist (DSM V, 20 item, 0 to 4 score)<br><u>Depression:</u><br>Mini-International Neuropsychiatric Interview (major depressive disorder, DSM IV)<br>Blinded application of questionnaire'<br>☐yes<br>☒no<br>☐not reported<br>Institution: University School of Medicine, New Haven, Connecticut Department of Psychiatry, Yale University School of Medicine, New Haven, Connecticut United States Department of Veterans Affairs National Center of Posttraumatic Stress Disorder, Clinical Neurosciences Division, West Haven, Connecticut United States

								Department of Veterans Affairs New England Mental Illness Research, Education, and Clinical Center, West Heven, Conneticut<br>Finance: US Department of Veterans Affairs National Centre for Posttraumatic Disorder, National Institute on Aging of the National Institute of Health
27	Tsuno and Kawakami 2016	Japan	Cross sectional study	Japan work stress and health Cohort Study (JSTRESS)<br>Employees of six Japanese manufacturing companies	No. of exposed:<br>58 female workers exposed to physical assaults at work<br>303 male workers exposed to physical assaults at work<br>No. of unexposed:<br>3,061 female workers not exposed to physical assaults at work<br>16,286 male workers not exposed to physical assaults at work<br>Response rate:<br>85% n.I. according to response rate in the aforementioned subgroups<br>Age (mean ± SD):<br>36.6 ± 10.6 years female employees<br>40.6 ± 9.02 years male employees<br>n.I. according to age in the aforementioned subgroups<br>Gender:<br>16.07% female, 83.93% male exp.<br>15.83% female, 84.17 % male unexp.	N.I.	Exposure:<br>Physical assaults at work according the NIOSH-Generic Job Stress Questionnaire<br>Duration of employment:<br>n.I.<br>Job duties:<br>n.I.	PTSD:<br>Not asked<br>Depression:<br>CES-D, Japanese version)<br>Blinded application of questionnaire'<br>☐yes<br>☒no<br>☐not reported<br>Institution: Institution: Wakayama Medical University<br>Finance: Ministry of Health, Labour and Welfare, Japan
28	van der Velden et al. 2013<br>'	Netherland	Cross sectional study	Police officers	No. of exposed:<br>144 police officers - group1 (east part of netherland)<br>503 police officers - group2 (involved in the Enschede disaster)<br>No. of unexposed:<br>114 Employees of banks, who were robbed – group 4<br>219 Employees of a psychiatric hospital –group 5<br>236 Soldiers before redeployment –group 8<br>123 firefighters –group 9<br>1,113 Employees of banks –group3	2002 Police officers group1<br>2004 Police officers group2<br>1991 Emplo-yees of banks – group 3<br>1999 Emplo-yees of banks – group 4	Exposure:<br>Police officers - group 1: n.I.<br>Police officers -group 2: involved in the Enschede disaster<br>Employees of banks, who were robbed group4<br>Duration of employment:<br>n.I.<br>Job duties:<br>n.I.	PTSD:<br>Not asked<br>Depression:<br>System Checklist Revised (SCL-90-R)-<br>Group 11 was interview by a different instrument (brief scale of SCL-90-R with 17 item)<br>Blinded application of questionnaire'<br>☐yes<br>☐no<br>☐not reported

					710 Employees who followed a training based on rational-motive therapy to strengthen their assertiveness group 6<br>278 Soldiers before deployment –group 7<br>76 Employees of a governmental social welfare organisation –group 10<br>335 Employees of supermarket –group 11<br><u>Response rate:</u><br>60% police officers -group 1<br>80.5% police officers -group 2<br>71% Employees of banks –group 3 +4<br>70% Employees of a psychiatric hospital group 5<br>74% Employees group 6<br>82.5% Soldiers group 7+8<br>48% firefigthers –group 9<br>65% Employees of a governmental organisation –group10<br>88% Employees of supermarket –group 11<br><u>Age (mean ± SD):</u><br>police officers -group 1<br>police officers -group 2<br>Employees of banks –group 3 +4<br>Employees of a psychiatric hospital group 5<br>Employees group 6<br>Soldiers group 7+8<br>firefighters –group 9<br>Employees of a governmental organisation –group10<br>Employees of supermarket –group 11<br><br><u>Gender:</u><br>13.9 % female 86.1% male police officers group1<br>11.3 % female 88.7% male police officers - group 2<br>65.9 % female 34.1% male Employees of banks group 3<br>52.7 % female 47.3% male Employees of banks group 4<br>62.1 % female 37.9% male Employees of a psychiatric hospital group 5<br>31.8 % female 68.2% male Employees group 6<br>12.2 % female 87.8% male Soldiers group 7	1996 employees of a psychiatric hospital – group 5<br>1997-1998 employees – group 6<br>2005-2007 soldiers group 7+8<br>2002 firefighter group9<br>1995 employees of a governmental organisation – group10<br>1996 employees of supermarket –group 11		Institution: Institute for Psychotrauma, Diemen, The Netherlands, Intervict, Tilburg University, Tilburg, The Netherlands, Arg Psychotrauma Expert Group, Diemen, The Netherlands, Military Mental Health Research Center and University Medical Utrecht, Utrecht, The Netherlands, National Institute for Public Health and the Environment, Biltho, The Netherlands<br>Finance: Dutch Ministry of Health, welfare and Sports, Saving Banks Association, the Netherlands<br>Institution of author/coauthor: Institute for Psychotrauma, Diemen, The Netherlands

					5.5 % female 94.5% male Soldiers group 8<br>3.3 % female 96.7% male firefighters –group 9<br>59.2 % female 40.8% male Employees of a g overnmental organisation –group10<br>43.9 % female 56.1% male Employees of supermarket –group 11			
29	Wieclaw et al. 2006	Denm ark	Matched Case control Study	Register based population data	<u>No. of cases:</u><br>3,007 persons (depression and other affective disorders)<br><u>No. of controls:</u><br>58,060 persons without depression matched for age, sex and time<br><u>Response rate:</u><br>100%(register based data)<br><u>Age:</u><br>18-65 years, mean and SD n.I., n.I. according to age in the aforementioned subgroups<br>matched for age<br><u>Gender:</u><br>n.I. according to gender in the aforementioned subgroups<br>matched for gender	N.I.	<u>Exposure:</u><br>job-exposure-matrix<br><u>Duration of employment:</u><br>n.I.<br><u>Job duties:</u><br>n.I.	<u>PTSD:</u><br>Not asked especially<br><u>Depression:</u><br>Clinical psychiatric diagnoses (ICD)<br>Blinded application of questionnaire'<br>☐yes<br>☐no<br>☐not reported<br>Institution: Aarhus University hospital<br>Finance: Danish Working environmental Council, Stanley Medical Search Institute, National Centre of registered-based research<br>Institution of author/co-author:
30	Wittchen et al. 2012	Germa ny	Cross sectional study	Soldiers deployed to Afghanistan war	<u>No. of exposed:</u><br>1483 soldiers who have been deployed to Afghanistan<br><u>No. of unexposed:</u><br>889 soldiers who have never been deployed oversees<br><u>Response rate:</u><br>92.8% soldiers in Afghanistan<br>95.4% soldiers who have never been oversees<br><u>Age (mean ±SD):</u><br>29.6 ± 7.3 years exp.<br>26.1 ± 6.7 years unexp.<br><u>Gender:</u><br>3 % female 97.0 % male exp.<br>12.4 % female 87.6 % male unexp.	2009/2010	<u>Exposure:</u><br>According the Combat Experiences Scale<br><u>Duration of employment:</u><br>n.I.<br><u>Job duties:</u><br>n.I.	<u>PTSD:</u><br>Composite International Diagnostic Interview (CIDI), DSM IV<br><u>Depression:</u><br>Not asked<br>Blinded application of questionnaire'<br>☐yes<br>☐no<br>☒not reported<br>Institution: Institute of Clinical Psychology and Psychotherapy & Center of Clinical Epidemiology and Longitudinal Studies, Technische Universität Dresden<br>Finance/funding: not reported

31	Zhao et al. 2018	China	Cross sectional study	Nurses	No. of exposed:<br>595 nurses experiencing workplace violence<br>No. of unexposed:<br>294 nurses not experiencing workplace violence<br>Response rate:<br>87.46 %, n.I. according to response rate in the aforementioned subgroups<br>Age (mean[1]):<br>31.26 years[1], SD n.I.<br>Gender:<br>97.5 % female 2.5 % male, n.I. according to gender in the aforementioned subgroups	2015-2016	Exposure:<br>Workplace violence scale<br>Duration of employment:<br>n.I.<br>Job duties:<br>n.I.	PTSD:<br>Not asked<br>Depression:<br>Self-rating depression scale (SDS)<br>Blinded application of questionnaire'<br>☒yes<br>☐no<br>☐not reported<br>Institution: Harbin Medical University<br>Finance: Harbin Medical University,

1 mean value own recalculation, [2] own calculation, exp.= exposed persons, unexp.= unexposed persons, n.I.=n.I.

Table S4 Effect estimates of studies included.

No.	Study (first author year)	*Outcome*	Effect Estimate				Number of cases		Risk estimate			Further analysis or information
			Prevalence, Incidence or mean	Exposed group or cases	Unexposed group or controls	Exposed group/ cases (n)	Exposed controls (n)	Unexposed group/controls (n)	RR, Or	Effect value (95% CI)	Adjusted for	
1	Ben Ezra et al. 2011	*PTSD*	Mean IES-R-Score<br>p=0.001 (study 1)<br>p=0.004 (study 2)	23.06<br>20.00	14.49<br>12.92	N.I.		N.I.	N.I.	N.I.	No adjustment<br>No significant differences between exposed and unexposed persons in terms of age, gender marital status, profession and income.	No infor-mation about number of cases or pre-valence , only mean score
		Depression	Mean CES-D-Score p=0.001 (study 1)<br>p=0.189 (study 2)	16.16<br>13.28	12.12<br>11.52	N.I.		N.I.	N.I.	N.I.		
2	Berg et al. 2006	*Depression*	Mean (SD) HADS-D-Score<br>*Female (age)*<br>20-29 years<br>30-39 years<br>40-49 years.<br>50-59 years.<br>*Male (age)*<br>20-29 years.<br>30-39 years<br>40-49 years.<br>50-59 years	1.6 (1.9)<br>2.5 (2.5)<br>3.0 (3.1)<br>3.2 (3.2)<br>1.5 (1.9)<br>2.8 (2.8<br>3.6 (3.1)<br>3.7 (3.2)	2.2 (2.4)<br>2.7 (2.8)<br>3.2 (3.0)<br>3.7 (3.1)<br>2.4 (2.4)<br>2.9 (2.7)<br>3.6 (3.0)<br>4.1 (3.2)	N.I.		N.I.	N.I.	N.I.		
3	Cothereau et al. 2004	*PTSD*	Prevalence for PTSD<br>directly after the accident<br>3month after the accident	4<br>1.6	0<br>0	8<br>8<br>3		0<br>0<br>0			No adjustment<br>No significant differences	Follow-up in exposed group (3month, 1year,

			1year after the accident	0.6	0	1		0			between exposed and control persons in terms of age, all drivers were male.	2years, 3years after acciden t
			2years after the accident	0.6	0	1		0				
			3years after the accident	0	0	0		0				
		Depressio n	Prevalence for severe depression			3		1				
			directly after the accident	1.5	0.5	3		1				
			3month after the accident	0	0	0		0				
			1year after the accident	0	0	0		0				
			2years after the accident	0	0	0		0				
			3years after the accident	0	0	0		0				
4	Darves-Bornoz et al. 2008	*PTSD*	12-Month PTSD prevalence When PTE Combat experience	8,4[1]	2,1[1]	18[1]		182[1]	OR PTSD when PTE (combat experience)	0.1 (0.0-0.3)	No adjustment N.I. about gender, age or exposure	
5	Feinstein et al. 2002	*PTSD*	Mean IES-R-Score	10.1	7.8	N.I.		N.I.			No adjustment<br><br>No significant differences between exposed and unexposed journalists to age and gender.	
			Prevalence SCID p=0.0001 (subsample 12/47)			Subsample : 12/47 cases		Subsample : 35/47 cases				
			Lifetime	28.6	0							
			Current	10.7	0	8		0				
			prewar	3.6	0	3		0				
						1		0				
		Depressio n	Mean BDI II-Score p=0.0001	10.1	7.8	N.I.		N.I.				
			GHQ 28 Subscale p=0.001	2.2	1.0	N.I.		N.I.				

			Prevalence SCID (subsample 12/47)<br>Lifetime<br>Current<br>prewar	 <br>21.4<br>7.1<br>3.6	 <br>5.3<br>0<br>5.3	Subsample 10/47 cases<br>6<br>2<br>1		Subsample 37/47 cases<br>1<br>0<br>1				
6	Feinstein 2012	*PTSD*	Mean IES-R-Score for intrusion<br>p= 0.07<br><br>Mean IES-R-Score for avoidance<br>p= 0.10<br><br>Mean IES-R-Score for hyper arousal<br>p= 0.17	1.67 (exp1)<br>1.10 (exp2)<br>1.63 (exp1)<br>0.98 (exp2)<br>1.62 (exp1)<br>0.28 (exp2)	1.05<br><br>1.11<br><br>0.59	N.I.		N.I.			No adjustment	No infor-mation about number of cases or Pre-valence, only mean score for exposed and unexposed group
		Depression	Mean BDI II-Score<br>p= 0.07<br><br>GHQ 28 Subscale<br>p= 0.12	1.73 (exp1)<br>1.16 (exp2)<br>20.0 (exp1)<br>13.18 (exp2)	1.02<br><br>14.65	N.I.		N.I.				
7	Hotopf et al. 2006	*PTSD*	Prevalence	3.8	4.3	Subsample 326/8525		subsample 100/2342	OR	0.61 (0.39-0.95)	Age, gender, rank, educational and marital status, service branch, fitness to deployed	
8	Huizink et al. 2006	*PTSD*	Prevalence<br>Firefighters<br><br>Police officers	 5.4<br><br>6.5	 2.6<br><br>2.4	 18[1]<br><br>54[1]		 5[1]<br><br>15[1]	OR	 1.1 (0.4-3.7)<br><br>2.8 (1.5-5.0)	Adjusted for age, gender, ethnicity, professional level, level of education, alcohol consumption,	

											smoking habits, level of physical activity, number of live events, and chronic diseases	
9	Kim et al. 2013	*PTSD*	Standardized prevalence ratios (SPRs)	2.1 (1.1-3.4) (all subway drivers)							Age, education, income, marital status, working career, economic difficulties	Only very few cases: number of PTDS cases (n=12) and number of Depres-sion cases (n=23) are very low
			**Lifetime prevalence** *p= 0.023*	3.0	0.7	8		4	**OR for lifetime** Ever PUT versus never	4.4 (1.3-16.4)		
			PUT was experienced over more than 1 year (n=229)	3.1	0.7	7		4	<u>When:</u> PUT was experienced over more than 1 year vs. never	4.4 (1.3-17.6)		
			PUT was experienced within 1 year (n=37) *p=0.030*	2.7	0.7	1		4	PUT was experienced within 1 year vs. never	3.4 (0.2-24.9)		
			Number of PUT =1 (n= 161)	3.7	0.7	6		4	<u>Number:</u> 1 PUT vs. never	5.1 (1.4-20.2)		
			Number of PUT ≥ 2 (n=105)	1.9	0.7	2		4	≥ 2 PUT vs. never::	2.6 (0.3-15.1)		
			Severity of victims injury not death (104)	4.8	0.7	5		4	<u>Severity of victims injury:</u>	5.7 (1.4-23.8)		

			death (n=162)	1.9	0.7	3		4	Not death vs. never<br><br>Death vs. never	2.9 (0.5-13.9)		
			**1-Year prevalence**<br>*p= 0.005*	2.3	0.2	6		1	**OR for 1 year**<br>Ever PUT versus never	11.7 (1.9-225,8)		
			PUT was experienced over more than 1 year (n=229)	2.2	0.2	5		1	<u>When:</u><br>PUT was experience d over more than 1 year vs. never	11.7 (1.8-229.7)		
			PUT was experienced within 1 year (n=37(<br>*p=0.006*	2.7	0.2	1		1	PUT was experience d within 1 year vs. never	12.0 (0.5-317.1)		
			Number of PUT =1 (n= 161)	2.5	0.2	4		1	<u>Number:</u><br>1 PUT vs. never	13.4 (1.9-265.3)		
			Number of PUT ≥ 2 (n=105)<br>*p=0.006*	1.9	0.2	2		1	≥ 2 PUT vs. never::	8.7 (0.7-201.7)		
			Severity of victims injury<br>not death (104)	3.9	0.2	4		1	<u>Severity of victims injury:</u>	17.9 (2.5-357.4)		
			death (n=162)	1.2	0.2	2		1		6.6 (0.6-147.3)		

			p=0.002						Not death vs. never<br><br>Death vs. never			
		Depression	Standardized prevalence ratios (SPRs)	0.8 (0.5-1.1) All subway drivers								
			**Lifetime prevalence**						**OR for lifetime** Major Depressive Disorder			
			p= n.s.	2.6	2.9	7		16	Ever PUT versus never	1.1 (0.4-2.7)		
			PUT was experienced over more than 1 year (n=229)	2.6	2.9	6		16	<u>When:</u> PUT was experienced over more than 1 year vs. never	1.1 (0.4-2.9)		
			PUT was experienced within 1 year (n=37)<br>*p=n.s.*	2.7	2.9	1		16	PUT was experienced within 1 year vs. never	0.9 (0.1-4.6)		
			Number of PUT =1 (n= 161)	2.5	2.9	4		16	<u>Number:</u> 1 PUT vs. never	0.9 (0.3-2.6),		
			Number of PUT ≥ 2 (n=105)	2.9	2.9	3		16	≥ 2 PUT vs. never::	1.5 (0.3-5.0)		
			Severity of victims injury	1.9	2.9	2		16				

			not death (104)						<u>Severity of victims injury:</u>	0.7 (0.1-2.6)		
			death (n=162)	3.1	2.9	5		16				
									Not death vs. never	1.4 (0.4-3.9)		
			**1-Year prevalence** *p= n.s.*	2.3	0.9	6		5	Death vs. never			
									**OR for 1 year**	2.6 (0.7-9.4)		
			PUT was experienced over more than 1 year (n=229)	2.2	0.9	5		5	Ever PUT versus never	2.5 (0.6-9.6)		
			PUT was experienced within 1 year (n=37(*p=n.s.*	2.7	0.9	1		5	<u>When:</u> PUT was experienced over more than 1 year vs. never	3.0(0.2-19.7)		
			Number of PUT =1 (n= 161)	2.9	0.9	4		5	PUT was experienced within 1 year vs. never	2.7(0.7-10.7)		
			Number of PUT ≥ 2 (n=105) *p=n.s.*	1.9	0.9	2		5				
									<u>Number:</u> 1 PUT vs. never	2.2 (0.3-11.9)		
			Severity of victims injury	1.0	0.9	1		5	≥ 2 PUT vs. never::	1.0(0.1-2.6)		
				3.1								

			not death (104)<br><br>death (n=162)<br>*p=n.s.*		0.9	5		5	Severity of victims injury:<br>Not death vs. never<br><br>Death vs. never	1.4 (0.4-3.9)		
10	Kim et al. 2014	*PTSD*	Standardized prevalence ratios (SPRs)	5.6 (3.1-8.8)<br>All subway drivers		N.I.		N.I.			Age	Except for age, other sozio-demographic factors were not associated with psychiatric disorders in univariate analysis
			**Lifetime Prevalence**	N.I.	N.I.	N.I.		N.I.	OR<br>PUT experience versus no experience<br><br>1 PUT experience versus 0 PUT experience<br><br>>= 2 PUT experience versus 0 PUT experience<br><br>PUT death versus PUT alive<br><br>When PUT was experienced vs. over 5 years	2.06 (0.94-4.55)<br><br>1.45 (0.55-3.85)<br><br>3.57 (1.32-3.65)<br><br>1.39 (0.40-4.82)<br><br>0.33		

			**1-Year Prevalence**	N.I.	N.I.	N.I.		N.I.	OR<br>PUT experience versus no experience<br><br>1 PUT experience versus 0 PUT experience<br><br>>= 2 PUT experience versus 0 PUT experience<br><br>PUT death versus PUT alive<br><br>When PUT was experienced vs. over 5 years	(0.03-2.63)<br><br>1.54 (0.52-4.55)<br><br>1.77 (0.31-4.47)<br><br>2.36 (0.57-9.70)<br><br>2.49 (0.27-23.27)<br><br>1.04 (0.11-9.06)	age	
		Depression	Standardized prevalence ratios (SPRs)	1.1 (0.7-1.7) All subway drivers		N.I.		N.I.				
			**Lifetime Prevalence**	N.I.	N.I.	N.I.		N.I.	OR		Age	

									PUT experience versus no experience<br><br>1 PUT experience versus 0 PUT experience<br><br>>= 2 PUT experience versus 0 PUT experience<br><br>PUT death versus PUT alive<br><br>When PUT was experienced vs. over 5 years	1.13 (0.55-2.31)<br><br>0.95 (0.45-2.25)<br><br>1.56 (0.56-4.34)<br><br>0.79 (0.25-2.53)<br><br>0.47 (0.09-2.46)		
			**1-Year Prevalence**	N.I.	N.I.	N.I.		N.I.	OR<br>PUT experience versus no experience<br><br>1 PUT experience versus 0	1.99 (0.72-5.53)<br><br>2.01 (0.66-6.11)	Age	

									PUT experience<br><br>>= 2 PUT experience versus 0 PUT experience<br><br>PUT death versus PUT alive<br><br>When PUT was experienced vs. over 5 years	1.94 (0.30-0.62)<br><br><br><br>0.59 (0.13-2.72)<br><br>1.04 (0.77-1.05)		
11	Kline et al. 2010	*PTSD*	Prevalence	20.96 (131/296)	8.64 (165/1910)	131		165	OR	2.70 (2.05-3.55)	Age, gender, race/ethnicity, education, income, marital status and deployment other than to Afghanistan or Iraq	
		Depression:	Prevalence	10.81 (32/296)	1.99 (38/1910)	32		38	OR for Major Depression exp. vs. unexp.	3.07 (1.81-5.19)		
12	Lam et al. 1999	*Depression*	Prevalence	n.I.	n.I.	n.I.		n.I.	OR unadj.<br><br>adj.	2.17 (1.32-3.55)<br><br>1.92 (1.14-3.22)	Stressful life event scale	

13	Lehavot et al. 2018	*PTSD*	Prevalence	N.I.	N.I.	N.I.		N.I.	OR<br>Women<br>Men	2.65 (0.83-8.41)<br>5.05 (3.60-7.10)	No adjustment	Sup-ple-mental mater-ial in the online version of the public-cation was not acces-sible under the given DOI-numbe r.
14	Levin-Rector et al. 2018	*PTSD:*	Prevalence	N.I.	N.I.	N.I.		N.I.	HR<br>Army soldiers per year of war deployme nt in the past<br>Marines per year of war deployme nt in the past	1.74 (1.71-1.76)<br>2.04 (1.93-2.15)	Multivariable model<br>days in service, marital status, cumulative years of deployment, gender, age, race, ethnicity, waiver status, rank, occupation, UIC-level suability	
		Depressio n:							HR	1.11		

									Army soldiers per year of war deployment in the past<br>Marines per year of war deployment in the past	(1.09-1.12)<br><br>1.12<br>(1.07-1.17)		
15	Luce et al. 2002	*PTSD*	Mean PSS-SR-Score (± SD)<br>Medical staff (n=33)<br><br>Occupational, speech and physio-therapists (n=27)<br><br>Managerial (n=20)<br><br>Administrative (n=52)<br>Nursing (n=301)<br><br>Social work (n=30)<br><br>Demestic/manual workers (n=34)<br><br>Home help/care (n=26)<br><br>Involved staff (total group) had	 6.21 (6.4)<br><br>7.30 (7.7)<br><br>7.95 (6.9)<br><br>9.38 (8.8)<br><br>11.09(9.6)<br><br>12.33(10.3)<br><br>15.41(13.8)<br><br>15.58 (13.4)	Controls (n=528) no involve-ment in the bombing	5.06 (6.8)					No adjustment<br>N.I. about age or gender	

			significantly higher PSS-SR-scores than controls (p<0.001)									
16	Madsen et al. 2010	*Use of Antide-pressants*	Prevalence<br>Threat of Violence<br><br>Physical Violence	 6.7<br><br>6.1	 6.9<br><br>6.9	 21<br><br>7		 321<br><br>335	OR<br>Threat of Violence<br><br>Physical Violence	 0.99 (0.63-1.58)<br><br>0.81 (0.31-1.76)	Gender, age, cohabitation, parental status, socioeconomic position	Expo-sure status was only obtain-ed at baselin e<br>Life events and other reasons for reactive depress -sion

												not assed at baseline and during follow-up
17	Magruder et al. 2005	*PTSD*	Prevalence	19.0	4.4	69		17	OR unadj,<br><br>adj,	5.05 (2.01-8.78)<br><br>9.08 (4.77-17.28)	Age, race, gender, site, education, war zone, living arrangement, employment status	59 of 86 persons with PTSD (68.6%) had comorbidity with major depression according MINI
18	Niedhammer et al. 2015	*Depression*	N.I.	N.I.	N.I.	N.I.		N.I.	ß Coefficient	Women: physical violence or sexual assault 0.17 (-0.29, 0.63)<br>Men: physical violence or sexual assault 1.09 (0.63, 1.55)	Adjusted for age, occupation and economic activity	

19	Oenning et al. 2018	*Depression*	N.I.	N.I.	N.I.	N.I.		N.I.	OR	work place violence: Women 2.625 (1.350-5.108) Men 3.299 (0.864-12.594)	Adjusted for sociodemographic factors, e.g. age, ethnicity and marital status and occupational factors, e.g. work status and economic activity	
20	Opie et al. 2010	*PTSD*	N.I.	N.I.	N.I.	N.I.		N.I.	Bivariate correlation	Sexual Harassment 0.21 (p<0.01) Sexual Abuse/ Assault 0.16 (p<0.01)	No adjustment	
21	Park 2011	*PTSD*	Prevalence	13.15	3.13	10		1	N.I.	N.I.	No adjustment	
22	Proctor et al. 1998	*PTSD*	Prevalence	5.8	0.0	12		0	N.I.	N.I.	No adjustment	The Ger-many cohort is signify-cantly older than the soldiers deplo-yed to Persian Gulf War.

												The prevalence of female soldiers in the Devens and New Orleans cohort is signifycantly higher as in the Germany cohort.
23	Rosellini et al. 2017	*PTSD*	Prevalence	23.5	4.0	996		848	OR	6.3 (5.7-6.9)	Adjusted for age and follow-up month	
24	Sareen et al. 2007	*PTSD*	N.I.	N.I.	N.I.	N.I.		N.I.	OR<br>soldiers exposed to combat<br><br>soldiers exposed to atrocities or massacers<br><br>soldiers exposed to peace-keeping	2.10 (1.28-3.45)<br><br>4.33 (2.79-6.72)<br><br>1.15 (0.71-1-86)	Adjusted for sex, age, marital status, income, education, military rank, type of forces, other deployment-related traumatic events	

		Depression	N.I.	N.I.	N.I.	N.I.		N.I.	OR<br>soldiers exposed to combat<br><br>soldiers exposed to atrocities or massacres<br><br>soldiers exposed to peace-keeping	1.36 (1.01-1.83)<br><br>1.82 (1.33-2.48)<br><br>0.95 (0.72-1.25)		
25	Sterud et al. 2008	*Depression*	Prevalence<br>Men<br>women	 8.0<br>4.6	 7.0<br>6.7	N.I.		N.I.	N.I.		Age	
26	Thomas et al. 2017	*PTSD*	Lifetime PTSD prevalence<br><br>Current PTSD prevalence	18.6<br>p < 0.001<br><br>11.1<br>p= 0.007	6.0<br><br>3.0	77<br><br>44		55<br><br>25	OR<br><br>OR	3.39 (2.22-5.16)<br><br>2.20 (1.23-3.89)	Age, sex, income, number of years in military, veterans affairs as primary source of health care, military branch, sum of traumatic life events	Combat group is signify-cantly younger than the non-exposed group
		Depression	Lifetime major depressive period<br><br>Current depression	13.6<br>p= 0.37<br><br>9.0<br>p= 0.82	8.8<br><br>5.9	58<br><br>48		79<br><br>46	OR<br><br>OR	1.19 (0.82-1.73)<br><br>1.05 (0.67-1.67)		
27	Tsuno and Kawakami 2016	*Depression*	N.I.	No informa-tion	No informa-tion	N.I.		N.I.	PR<br><br>male	 <br>2.18 (1.94-2.45)	Age, education and marital status	

									female	1.63 (1.24-2.13)		
28	van der Velden et al. 2013	Very severe depressive symptoms	Prevalence	Police officer group 1: 1.4<br>Police officer group 2: 1.2	Bank employ-ees: 2.7<br>Soldiers: 0.4<br>Social welfare: 5.3	Police officer group 1: 2<br>Police officer group 2: 6		Bank employees: 30<br>Soldiers: 1<br>Social welfare: 4	OR	Police officer group 1: 1 (Reference)<br>Police officer group 2: 0.86 (0.17-4.32)<br>Bank employees: 2.09 (0.48-9.03)<br>Soldiers: 0.22 (0.02-2.52)<br>Social welfare: 4.63 (0.81-26.49)	Adjusted for age, sex and education	
29	Wieclaw et al. 2006	*Depression*				women<br>**Threats** at work 0% (Reference) 905<br>Low >0-≤ 20% 1,862<br>High >20 % 240<br>**Violence** at work<br>0%<br>(Reference)<br>1,415	women<br>3,756<br>7,165<br>765<br>6,009		OR	1.00<br>1.14 (1.04-1.26)<br>1.48 (1.23-1.79)<br>1.00	Matched for age, sex and time<br>Marital status, having children, level of education, income level, total level of unemployment, residence and nationality	Out-come aren`t mea-sured specifi-cly for PTSD within "stress related disor-ders"

						Low >0-≤ 20% 1,173<br><br>High >20 % 419	4,468<br><br>1,209			1.25 (1.03-1.23)<br><br>1.45 (1.27-1.65)		
						<u>men</u><br>**Threats** at work 0% (Reference) 892<br>Low >0-≤ 20% 1,044<br><br>High >20 % 105<br>**Violence** at work 0% (Reference) 1,558<br>Low >0-≤ 20% 356<br><br>High >20 % 127	<u>men</u><br><br>4,100<br><br>4,256<br><br>408<br><br>6,893<br><br>1,513<br><br>358		OR	1.00<br><br>1.07 (0.96-1.19)<br><br>1.17 (0.92-1.48)<br><br>1.00<br><br>1.03 (0.90-1.18)<br><br>1.48 (1.18-1.86)		
30	Wittchen et al 2012	*PTSD*	Prevalence<br>All<br>Combat unit	2.9<br>2.6	1.2	43<br>19		22	OR	2.5 (1.1-5.6)<br>3.3 (0.5-23.7)	N.I.	
31	Zhao et al. 2018	*Depression*	Prevalence	No information	No information	N.I.		N.I.	ß	0.110 (p<0.01)	Adjusted for age, gender, education, marital status, professional	

1 mean value own recalculation, ² own calculation, exp.= exposed persons, unexp.= unexposed persons, N.I. no Information

Figures S2.-S9. *Meta-Analysis Results.*

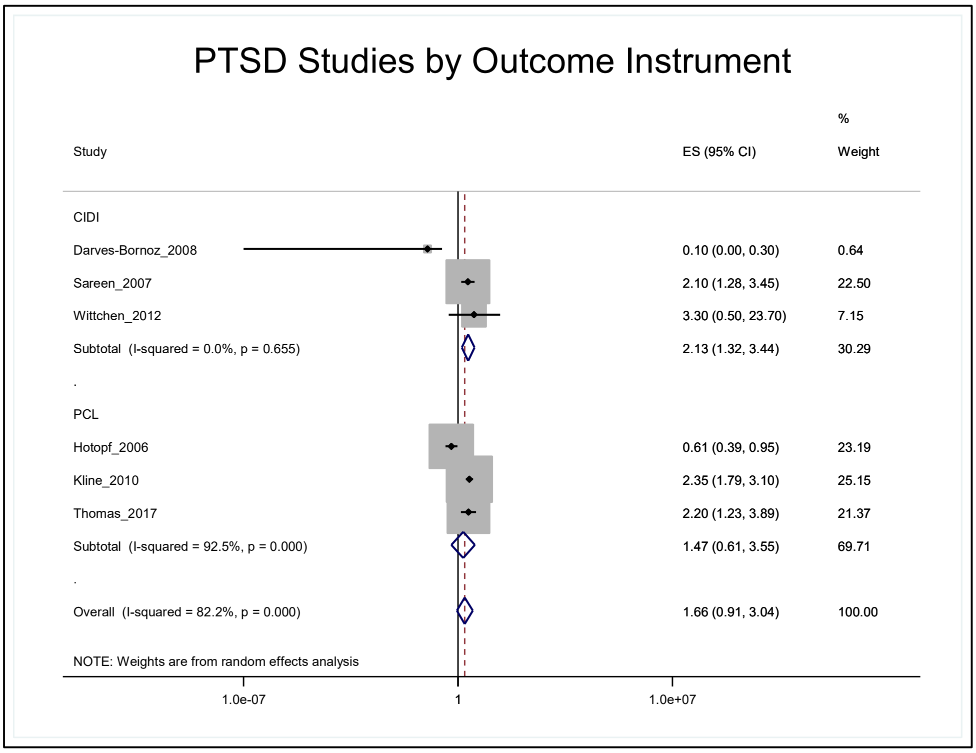

Figure S2. *Studies of PTSD in soldiers after war deployment, studies by outcome instrument.*

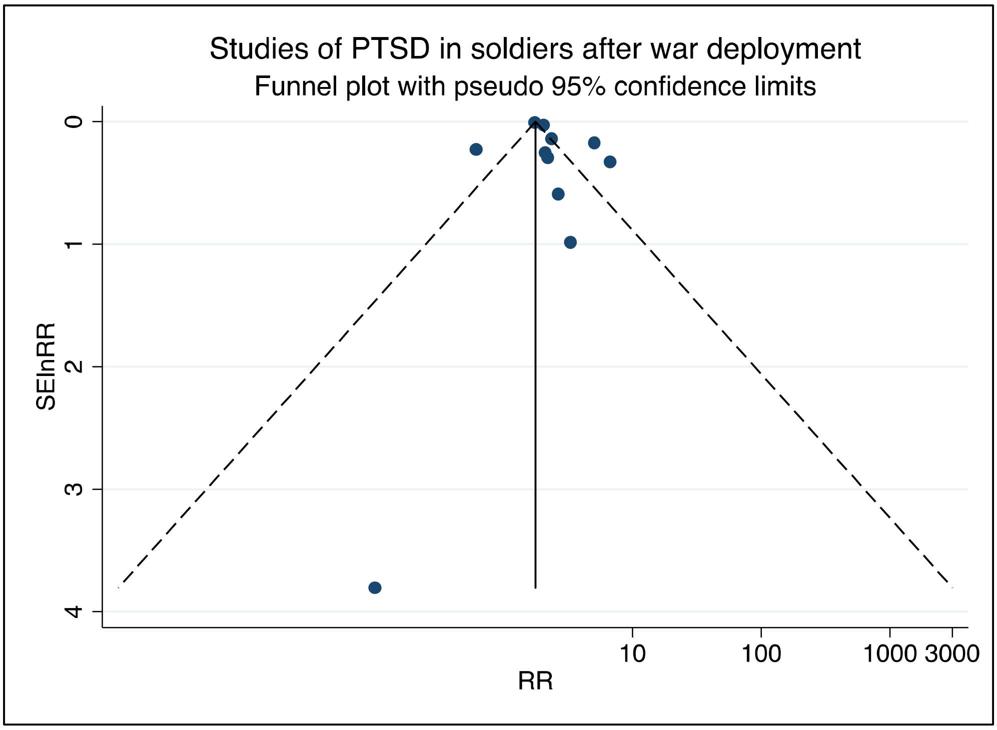

Egger's test: p=0.21

Figure S3. *Studies of PTSD in soldiers after war deployment: funnel plot with pseudo 95% confidence limits.*

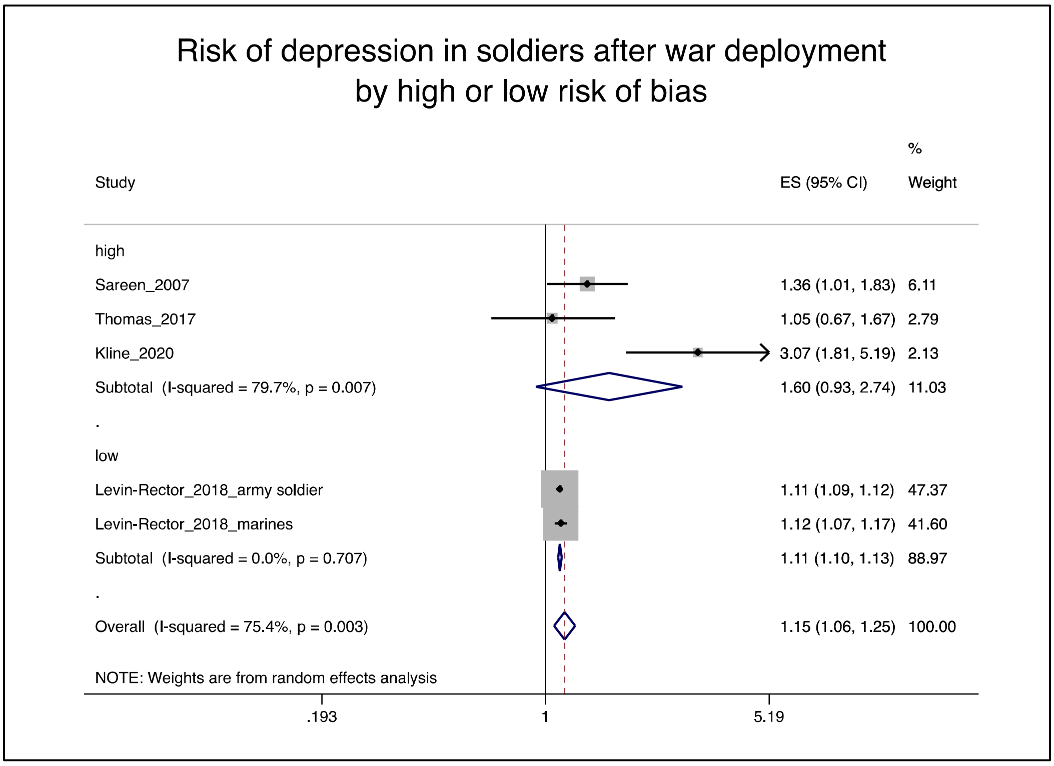

Figure S4. *Risk of depression in soldiers after war deployment by high or low risk of bias.*

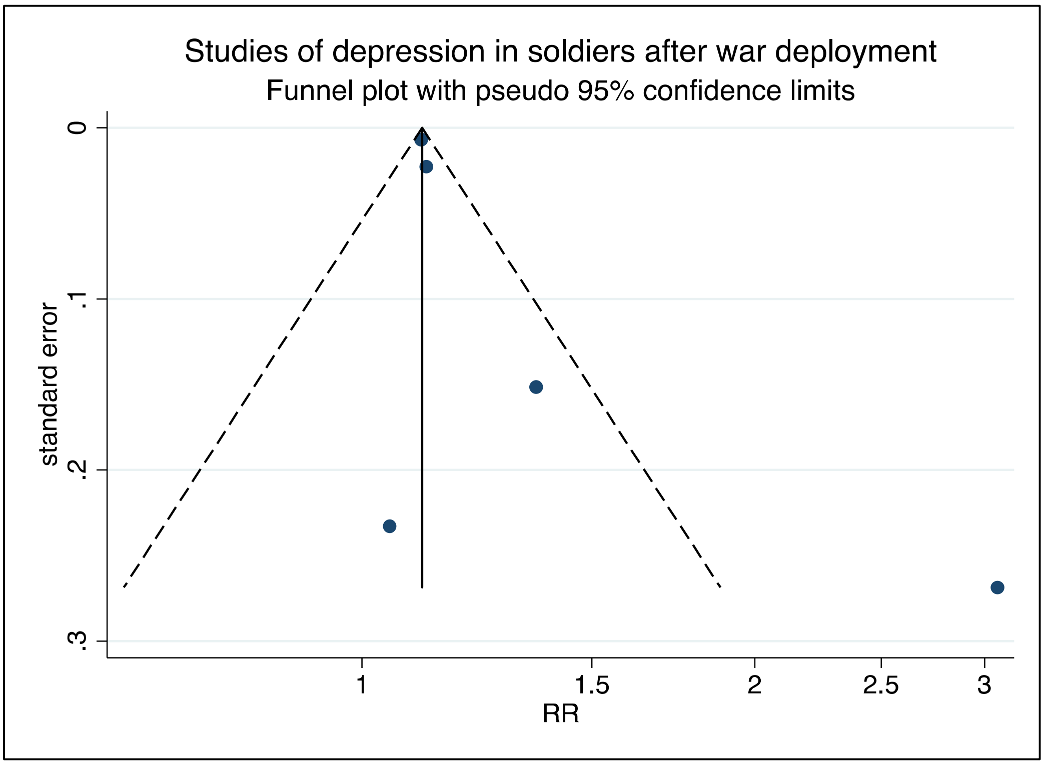

Egger's test: p= 0.20

Figure S5. *Risk of depression in soldiers after war deployment: funnel plot with pseudo 95% confidence limits.*

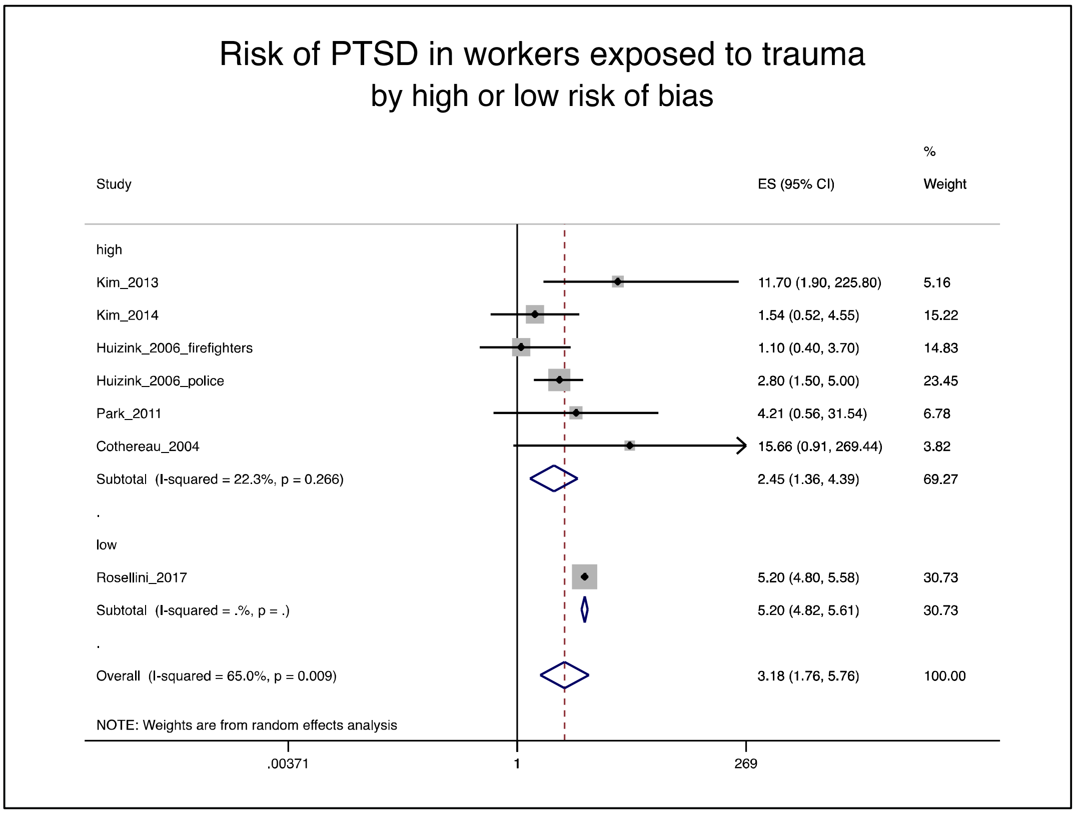

Figure S6. *Risk of PTSD in workers exposed to trauma by high or low risk of bias.*

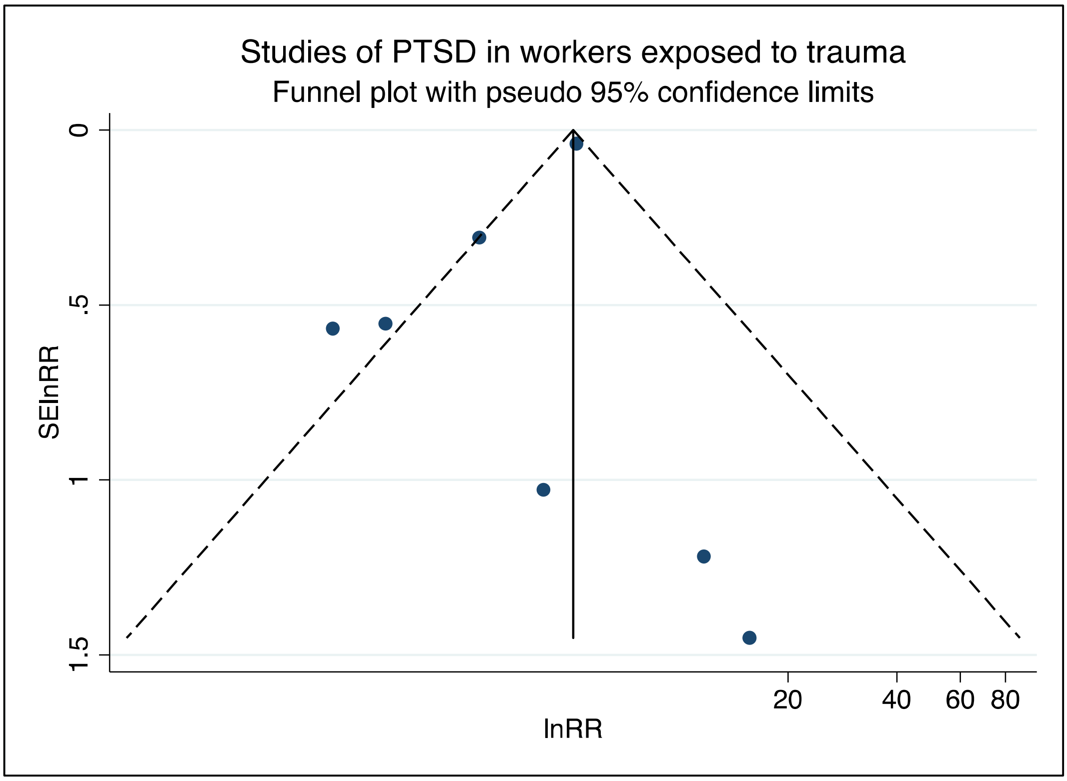

Egger's test: P= 0.223

Figure S7. *Risk of PTSD in workers exposed to trauma, funnel plot with pseudo 95% confidence limits.*

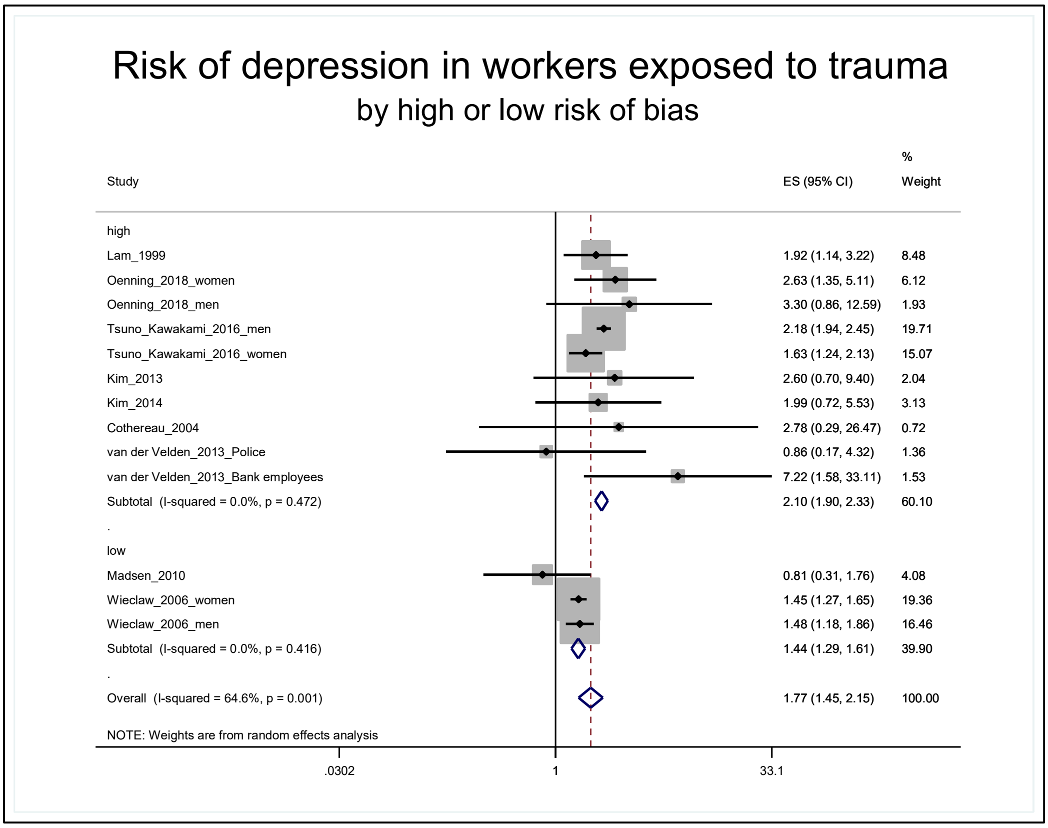

Figure S8. *Risk of depression in workers exposed to trauma by high or low risk of bias.*

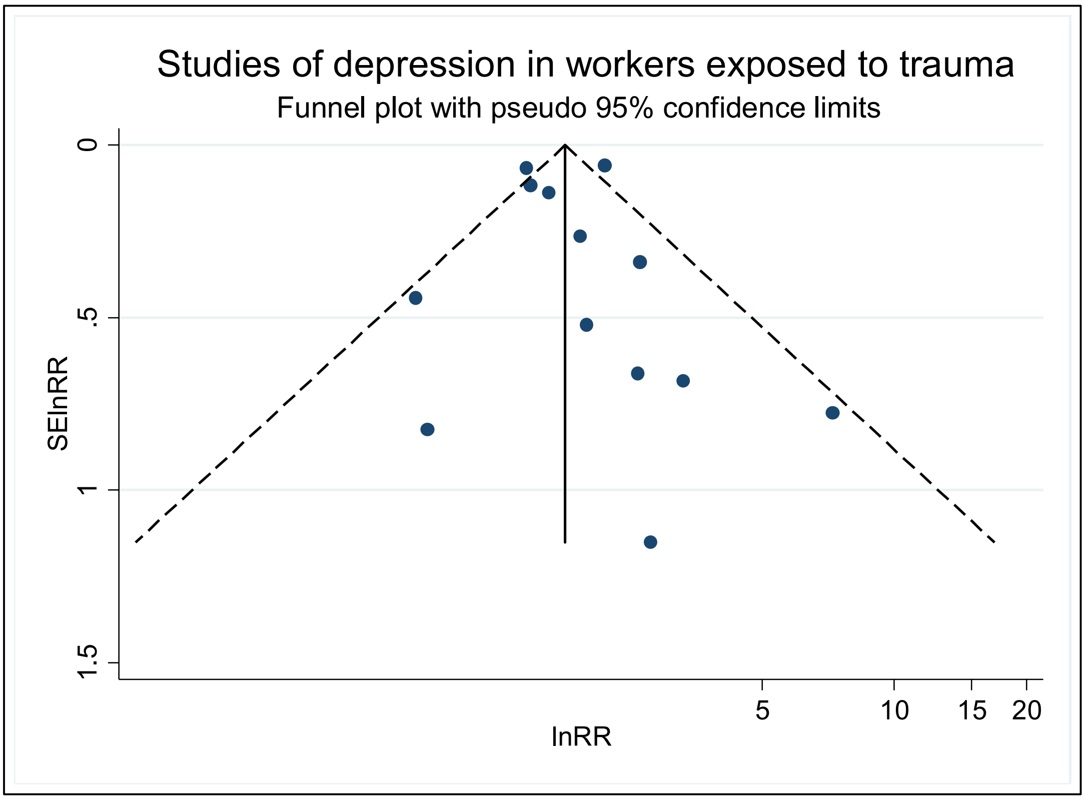

Egger's test: p=0.83

Figure S9. *Risk of depression in workers exposed to trauma, funnel plot with pseudo 95% confidence limits.*
